# Million Hearts: Prevalence of Leading Cardiovascular Disease Risk Factors — United States, 2005–2012

**Published:** 2014-05-30

**Authors:** Matthew D. Ritchey, Hilary K. Wall, Cathleen Gillespie, Mary G. George, Ahmed Jamal

**Affiliations:** 1 Division for Heart Disease and Stroke Prevention; 2 Office on Smoking and Health, National Center for Chronic Disease Prevention and Health Promotion, CDC

Each year, approximately 1.5 million U.S. adults have a heart attack or stroke, resulting in approximately 30 deaths every hour and, for nonfatal events, often leading to long-term disability (1). Overall, an estimated 14 million survivors of heart attacks and strokes are living in the United States (1). In 2011, the U.S. Department of Health and Human Services, in collaboration with nonprofit and private organizations, launched Million Hearts (http://www.millionhearts.hhs.gov), an initiative focused on implementing clinical and community-level evidence-based strategies to reduce cardiovascular disease (CVD) risk factors and prevent a total of 1 million heart attacks and strokes during the 5-year period 2012–2016 (2,3). From 2005–2006 to the period with the most current data, analysis of the Million Hearts four “ABCS” clinical measures (for aspirin, blood pressure, cholesterol, and smoking) showed 1) no statistically significant change in the prevalence of aspirin use for secondary prevention (53.8% in 2009–2010), 2) an increase to 51.9% in the prevalence of blood pressure control (in 2011–2012), 3) an increase to 42.8% in the prevalence of cholesterol management (in 2011–2012), and 4) no statistically significant change in the prevalence of smoking assessment and treatment (22.2% in 2009–2010). In addition, analysis of two community-level indicators found 1) a decrease in current tobacco product smoking (including cigarette, cigar, or pipe use) prevalence to 25.1% in 2011–2012 and 2) minimal change in mean daily sodium intake (3,594 mg/day in 2009–2010). Although trends in some measures are encouraging, further reductions of CVD risk factors will be needed to meet Million Hearts goals by 2017.

## Data Sources

Data from the National Health and Nutrition Examination Survey (NHANES*) for 2005–2012 were used to calculate prevalence estimates for managed low-density lipoprotein cholesterol (LDL-C†) among hyperlipidemic adults aged ≥20 years and estimates for controlled blood pressure§ among hypertensive adults aged ≥18 years. The 2005–2010 NHANES data were the most recent available to estimate the mean daily sodium intake (mg/day¶) among adults aged ≥18 years. Data from the 2005–2010 National Ambulatory Medical Care Survey (NAMCS**) were combined into 2-year cycles to estimate the prevalence of office visits to primary care physicians and cardiologists where aspirin or other antiplatelet medication was prescribed to adults aged ≥18 years with ischemic vascular disease.†† Additionally, NAMCS data were used to estimate the prevalence of office visits where smoking treatment was prescribed among adults aged ≥18 years who were identified as current tobacco users.§§ Data from the 2005–2012 National Survey on Drug Use and Health (NSDUH¶¶) were combined into 2-year cycles to estimate the prevalence of current tobacco product smoking*** among adults aged ≥18 years. This newly adopted measure of current tobacco smoking has been included because it measures all combustible tobacco product use, which is a major CVD risk factor (1) and not just cigarette use, as was the case with previous measures.

Up to four survey cycles (2005–2006, 2007–2008, 2009–2010, and 2011–2012) were examined using sex-, age-, and race/ethnicity-adjusted linear trends analyses (p<0.05). Sex-, age-, and race/ethnicity-adjusted t-tests were used to examine 1) prevalence changes comparing the two most recent data cycles (p<0.05) and 2) differences between sex, age, and race-ethnicity groups within the most recent data cycle (p<0.05).

## ABCS Clinical Measures

**FIGURE 1:**
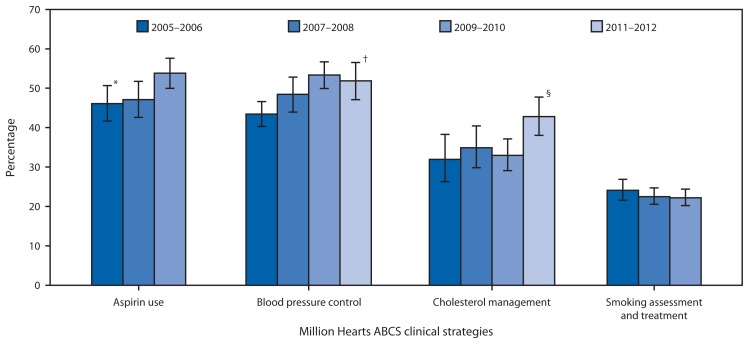
Prevalence of Million Hearts “ABCS” clinical strategies to prevent cardiovascular disease among adults — United States, 2005–2006 to 2011–2012 **Abbreviation:** ABCS = aspirin use for secondary prevention, blood pressure control, cholesterol management, smoking assessment and treatment. * 95% confidence interval. ^†^ Linear trend adjusted for sex, age group, and race/ethnicity was statistically significant from 2005–2006 through 2011–2012 (p<0.05). ^§^ Difference between 2009–2010 and 2011–2012 is statistically significant (p<0.05).

In 2009–2010, prevalence of recommended aspirin use was greater among men (58.5%) than women (48.0%) and greater among non-Hispanic whites (55.7%) compared with Hispanics (43.6%) (Table 1). The prevalence of blood pressure control improved from 43.4% in 2005–2006 to 51.9% in 2011–2012 (Figure 1); in 2011–2012, the prevalence was greater among women (54.6%) than men (48.9%) and greater among adults aged 45–64 years (56.3%) compared with those aged 18–44 (42.2%) and ≥75 years (41.7%).

The prevalence of cholesterol management increased from 33.0% in 2009–2010 to 42.8% in 2011–2012 (Figure 1); in 2011–2012, the prevalence was greater among adults aged 65–74 years (59.6%) and lower among those aged 20–44 (11.6%) compared with those aged 45–64 years (44.1%) (Table 1). Additionally, the prevalence was higher among non-Hispanic whites (47.4%) compared with non-Hispanic blacks (35.5%) and Hispanics (23.0%). In 2009–2010, the prevalence of smoking assessment and treatment (e.g., cessation medication or counseling) was greater among adults aged 45–64 years (25.3%) compared with those aged 18–44 (20.0%) and ≥65 years (18.9%).

## Community-Level Risk Factor Measures

**FIGURE 2:**
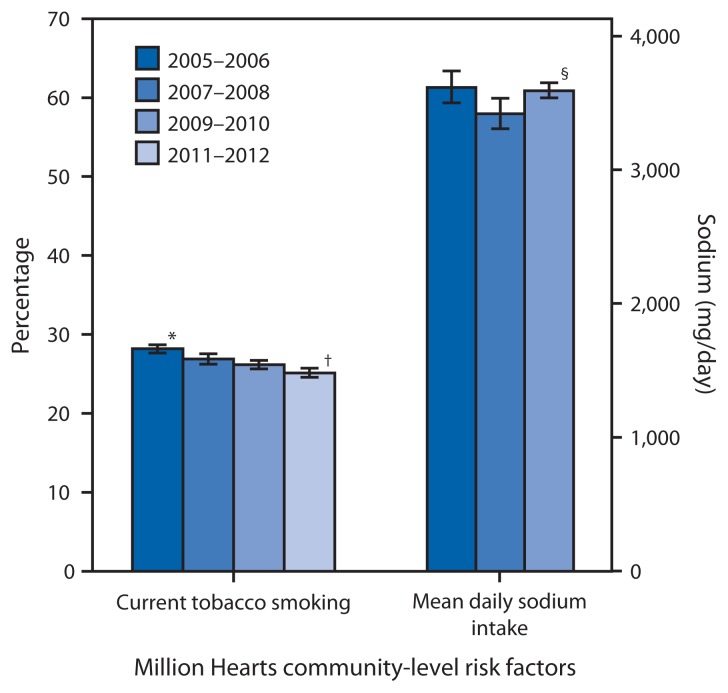
Values for Million Hearts community-level risk factors for cardiovascular disease among adults — United States, 2005–2006 to 2011–2012 * 95% confidence interval. ^†^ Linear trend adjusted for sex, age group, and race/ethnicity was statistically significant from 2005–2006 through 2011–2012 (p<0.05). ^§^ Linear trend adjusted for sex, age group, and race/ethnicity was statistically significant from 2005–2006 through 2009–2010 (p<0.05).

Current tobacco product (cigarettes, cigars, or a pipe) smoking prevalence decreased from 28.2% in 2005–2006 to 25.1% in 2011–2012 (Figure 2). This 11% decline corresponded with a decrease of 11% in current cigarette smoking prevalence from 20.9% in 2005–2006 to 18.5% in 2011–2012, measured using National Health Interview Survey data.††† In 2011–2012, current tobacco product smoking was greater among men (30.3%) than women (20.4%), adults aged 18–44 years (30.5%) compared with those aged 45–64 (24.6%) or ≥65 years (11.4%), and non-Hispanic whites (27.1%) compared with non-Hispanic blacks (26.2%) and Hispanics (18.1%) (Table 2).

The mean daily sodium intake decreased slightly from 3,619 mg/day in 2005–2006 to 3,594 mg/day in 2009–2010 (Figure 2). The most current data show mean daily sodium intake was greater among men (4,225 mg) than among women (2,976 mg), greater among adults aged 18–44 years (3,770 mg) compared with those aged 45–64 (3,640 mg) and ≥65 years (2,992 mg), and greater among non-Hispanic whites (3,631 mg) compared with non-Hispanic blacks (3,352 mg) and Hispanics (3,431 mg) (Table 2).

### Discussion

To reach the goal of preventing 1 million heart attacks and strokes during 2012–2016, Million Hearts set population-level goals of achieving ≥65% prevalence for each ABCS clinical measure as well as a 20% reduction in sodium intake (to approximately 2,900 mg/day) and a 10% reduction in current tobacco product smoking prevalence (to approximately 23.6%) (2). A goal to decrease mean daily trans-fatty acid intake is still being promoted (e.g., by supporting ongoing efforts to remove artificial trans-fats from the food supply); however, regular measurement has been deemphasized because of the considerable recent decreases in trans-fat consumption (4) and the cost of regularly obtaining population estimates of consumption. Million Hearts has focused on improving performance in specific clinical and community-level CVD risk factors because interventions in these areas have been shown to be effective ways to greatly decrease CVD morbidity and mortality (2).

Current estimates from 2005–2012 for certain Million Hearts measures serve as baseline values for achieving the initiative’s 2017 goals. Additional progress needs to be made in all reported measures important to cardiovascular health, especially among those groups with the smallest prevalence of desired characteristics. For example, the most recently available data show that, compared with those aged ≥45 years, younger adults were more likely to have uncontrolled blood pressure and poorly managed cholesterol, and to smoke tobacco products; younger adults were less likely to receive smoking assessment and treatment, and had greater mean daily sodium intakes. These differences place younger adults at considerable risk for developing CVD and suffering a CVD-related event during their lifetime; persons with two or more major CVD risk factors by age 50 years have more than 10 times the risk for developing atherosclerotic CVD compared with those who are free from major CVD risk factors at that age (5).

The findings in this report are subject to at least seven limitations. First, new cholesterol management guidelines recently released by the American College of Cardiology (ACC) and American Heart Association (AHA) focus on providing treatment with appropriate types and doses of cholesterol-lowering medications (statins) rather than routine treatment to cholesterol targets (6). The cholesterol management rates reported here are based on the previous guidelines in place when the data were collected and the initiative was launched. Second, debate continues over what population-level thresholds should be used to demonstrate adequate blood pressure control, particularly among older adults (7). This report uses the thresholds recommended for the general population by the Seventh Joint National Committee on Prevention, Detection, Evaluation, and Treatment of High Blood Pressure, because the recommendations remain endorsed by organizations including the ACC, AHA, and the National Institutes of Health and aligns with *Healthy People 2020* measures. Third, response rates for the three different surveys ranged from 58.3% to 77.4%, and the results might be subject to nonresponse bias. Fourth, each survey used excludes certain population segments. For example, NHANES surveys include only the noninstitutionalized U.S. population and do not include military personnel. Fifth, one of the smoking cessation medications, bupropion, has multiple indications; however, all bupropion prescriptions were considered as cessation treatment, representing approximately 10% of all documented cessation interventions. Sixth, NAMCS-based visit estimates rely on health-care providers’ intervention documentation, for which the quality might vary over time, thereby affecting trend analyses. Finally, the aspirin measure describes the health-care provider’s recommended use of aspirin or other antiplatelet medication at a visit and not actual medication use; the indication for use is also not collected. Measures of patient-reported aspirin use are being explored.

Million Hearts strategies (2,3,8) that address these CVD risk factors include promoting use of standardized hypertension treatment protocols (9), effective use of health information technology (2), and self-measured blood pressure monitoring with clinical support.§§§ Other strategies that Million Hearts supports include the following: use of CVD-related clinical quality measures and their incorporation into quality reporting initiatives (10); supporting the Tips From Former Smokers campaign¶¶¶; comprehensive smoke-free policy adoption; implementation of The Community Preventive Services Task Force recommendations, including use of team-based care and reduction of out-of-pocket prescription medication costs****; and population dietary sodium reduction efforts.†††† Additional focus on both clinical-level efforts that support consistent and coordinated patient care and community-level efforts that promote environments that encourage healthy behaviors and reduce unhealthy exposures is needed to continue progress towards meeting Million Hearts goals by 2017.

What is already known on this topic?

Approximately 1.5 million U.S. adults have a heart attack or stroke each year. These events often lead to long-term disability or death. In 2011, the U.S. Department of Health and Human Services, in collaboration with other key partners, launched Million Hearts, an initiative focused on implementing clinical and community evidence-based strategies to prevent 1 million heart attacks and strokes for the 5-year period 2012–2016.

What is added by this report?

From 2005–2006 to the period with the most current data, prevalence of the Million Hearts “ABCS” of clinical care showed no significant change for aspirin use for secondary prevention (53.8% in 2009–2010), improved to 51.9% for blood pressure control and to 42.8% for cholesterol management (in 2011–2012), and showed no significant change for smoking assessment and treatment (22.2% in 2009–2010). Analysis of two community-level indicators found a decrease in current tobacco product (cigarettes, cigars, or a pipe) smoking prevalence to 25.1% (in 2011–2012) and minimal change in mean daily sodium intake (3,594 mg/day in 2009–2010).

What are the implications for public health practice?

Although trends in some measures are encouraging, additional efforts to reduce cardiovascular risk factors are needed to meet the 2017 Million Hearts goals.

## Figures and Tables

**TABLE 1:** Current prevalence of implementation of Million Hearts “ABCS” clinical strategies to prevent cardiovascular disease among adults — United States, 2009–2010, 2011–2012

Clinical strategy	%*	(95% CI)	p-value using adjusted t-test†
**Aspirin use for secondary prevention (2009–2010)** §			
**Total**	**53.8**	**(50.0–57.6)**	**—**
Men	58.5	(54.1–62.9)	referent
Women	48.0	(42.8–53.3)	0.001
**Age group (yrs)**			
18–44	38.5	(22.4–57.4)	0.213
45–64	54.1	(47.9–60.2)	referent
≥65	54.5	(50.5–58.5)	0.636
65–74	58.9	(52.4–65.0)	0.159
≥75	51.4	(46.6–56.2)	0.681
**Race/Ethnicity**			
White, non–Hispanic	55.7	(51.5–59.9)	referent
Black, non–Hispanic	50.4	(37.9–62.9)	0.700
Hispanic	43.6	(36.3–51.1)	0.012
Other	52.5	(41.3–63.5)	0.588
**Blood pressure control (2011–2012)** ¶			
**Total**	**51.9**	**(47.1–56.6)**	**—**
Men	48.9	(44.4–53.5)	referent
Women	54.6	(48.5–60.5)	0.017
**Age group (yrs)**			
18–44	42.2	(32.0–53.2)	0.032
45–64	56.3	(49.6–62.8)	referent
≥65	50.1	(45.0–55.2)	0.032
65–74	57.9	(51.0–64.4)	0.802
≥75	41.7	(33.5–50.5)	0.001
**Race/Ethnicity**			
White, non–Hispanic	53.9	(47.6–60.1)	referent
Black, non–Hispanic	48.7	(43.1–54.3)	0.124
Hispanic	45.9	(38.6–53.4)	0.140
Other	46.0	(35.4–56.9)	0.324
**Cholesterol management (2011–2012)** **			
**Total**	**42.8**	**(38.0–47.7)**	**—**
Men	40.9	(35.4–46.8)	referent
Women	44.8	(37.9–51.9)	1.000
**Age group (yrs)**			
20–44	11.6	(6.0–21.0)	<0.001
45–64	44.1	(38.3–50.2)	referent
≥65	56.7	(49.8–63.4)	0.004
65–74	59.6	(48.3–69.9)	0.015
≥75	52.2	(38.2–65.8)	0.350
**Race/Ethnicity**			
White, non–Hispanic	47.4	(41.3–53.6)	referent
Black, non–Hispanic	35.5	(28.7–43.0)	0.034
Hispanic	23.0	(16.1–31.8)	0.001
Other	43.2	(29.2–58.4)	0.950
**Smoking assessment and treatment (2009–2010)** ††			
**Total**	**22.2**	**(20.2–24.4)**	**—**
Men	21.1	(18.8–23.6)	referent
Women	23.2	(20.4–26.2)	0.157
**Age group (yrs)**			
18–44	20.0	(17.1–23.3)	0.003
18–24	17.3	(12.6–23.3)	0.006
25–44	20.6	(17.6–24.0)	0.011
45–64	25.3	(22.5–28.3)	referent
≥65	18.9	(15.7–22.5)	0.002
65–74	20.0	(16.2–24.4)	0.025
≥75	16.5	(11.1–24.0)	0.031
**Race/Ethnicity**			
White, non–Hispanic	21.9	(19.6–24.4)	referent
Black, non–Hispanic	25.9	(19.7–33.3)	0.237
Hispanic	22.7	(16.7–30.1)	0.685
Other	15.0	(8.5–25.2)	0.190

**Abbreviations:** ABCS = aspirin use for secondary prevention, blood pressure control, cholesterol management, smoking assessment and treatment; CI = confidence interval.

* Weighted, unadjusted estimates.

† t-test for statistically significant differences among demographic subgroups, adjusted for sex, age group, and race/ethnicity, using linear/logistic regression.

§ Source: National Ambulatory Medical Care Survey (NAMCS). Includes office visits to primary care physicians and cardiologists by patients aged ≥18 years with ischemic vascular disease in which aspirin or other antiplatelet medications are prescribed. Excludes visits by patients with a contraindicated condition or medication and obstetric and gynecologic visits.

¶ Source: National Health and Nutrition Examination Survey (NHANES). Blood pressure (BP) control is defined as an average systolic BP <140 mmHg and an average diastolic BP <90 mmHg. Calculated among adults aged ≥18 years with hypertension. Hypertension defined as an average systolic BP ≥140 mmHg, or an average diastolic BP ≥90 mmHg, or self-reported current use of BP-lowering medication, defined as an answer of “yes” to the following questions: “Because of your high blood pressure/hypertension, have you ever been told to take prescribed medicine?” and “Are you currently taking medication to lower your blood pressure?” Excludes pregnant women.

** Source: NHANES. Cholesterol control is defined as a fasting low-density lipoprotein cholesterol (LDL-C) value among adults aged ≥20 years below the target levels (<100 mg/dL for the high risk group, <130 mg/dL for the intermediate risk group, and <160 mg/dL for the low risk group). Calculated among those with LDL-C dyslipidemia, defined using National Cholesterol Education Program’s Adult Treatment Panel III risk categories based on the risk for developing coronary heart disease in the next 10 years. Additional information available at http://www.nhlbi.nih.gov/guidelines/cholesterol/index.htm. Current use of cholesterol-lowering medication is defined as an answer of “yes” to the following questions: “To lower your blood cholesterol have you ever been told by a doctor or other health professional to take prescribed medicine?” and “Are you now following this advice to take prescribed medicine?” Excludes pregnant women.

†† Source: NAMCS. Includes physician office visits by persons aged ≥18 years who screened positive for current tobacco use during which tobacco cessation counseling or cessation medications were provided. Additional stratification provided for adults aged 18–24 and 25–44 years because of higher prevalence of tobacco use among these age groups.

**TABLE 2:** Current values for Million Hearts community-level risk factors for cardiovascular disease among adults — United States, 2009–2010, 2011–2012

Community-level risk factor	(%*)	(95% CI)	p-value using adjusted t-test†
**Current tobacco product smoking (2011–2012)** §			
**Total**	**25.1**	**(24.6–25.7)**	**—**
Men	30.3	(29.4–31.1)	referent
Women	20.4	(19.7–21.0)	<0.001
**Age group (yrs)**			
18–44	30.5	(29.3–31.1)	<0.001
18–24	31.2	(30.5–31.9)	<0.001
25–44	30.2	(29.5–31.0)	<0.001
45–64	24.6	(27.5–25.5)	referent
≥65	11.4	(10.4–12.5)	<0.001
65–74	15.3	(13.8–16.9)	<0.001
≥75	5.7	(4.6–7.1)	<0.001
**Race/Ethnicity**			
White, non-Hispanic	27.1	(26.4–27.9)	referent
Black, non-Hispanic	26.2	(24.7–27.8)	0.004
Hispanic	18.1	(16.9–19.2)	<0.001
Other	19.2	(17.5–21.1)	<0.001
**Community-level risk factor**	**(Mean** * **)**	**(95% CI)**	**p-value using adjusted t-test**

**Daily sodium intake (mg/day) (2009–2010)** ¶			
**Total**	**3,594**	**(3,537–3,651)**	**—**
Men	4,255	(4,167–4,342)	referent
Women	2,976	(2,920–3,032)	<0.001
**Age group (yrs)**			
18–44	3,770	(3,702–3,837)	0.025
18–24	3,749	(3,559–3,940)	0.320
25–44	3,777	(3,688–3,866)	0.033
45–64	3,640	(3,542–3,739)	referent
≥65	2,992	(2,879–3,106)	<0.001
65–74	3,175	(3,061–3,289)	<0.001
≥75	2,741	(2,591–2,891)	<0.001
**Race/Ethnicity**			
White, non-Hispanic	3,631	(3,564–3,698)	referent
Black, non-Hispanic	3,352	(3,233–3,471)	0.001
Hispanic	3,431	(3,332–3,530)	<0.001
Other	3,994	(3,663–4,324)	0.054

**Abbreviation:** CI = confidence interval.

* Weighted, unadjusted estimates.

† t-test for statistically significant differences among demographic subgroups, adjusted for sex, age group, and race/ethnicity, using linear/logistic regression.

§ Source: National Survey on Drug Use and Health. Includes current use of combustible tobacco products (i.e., cigarettes, cigars, or pipes) among adults aged ≥18 years. Current cigarette smoking defined as an answer of “yes” to the question, “Have you smoked at least 100 cigarettes in your entire life?” and an answer of “Within the past 30 days” to the question “How long has it been since you last smoked part or all of a cigarette?” Current cigar smoking is defined as an answer of “Within the past 30 days” to the question, “How long has it been since you last smoked part or all of any type of cigar?” Current pipe smoking is defined as an answer of “yes” to the question, “During the past 30 days, have you smoked tobacco in a pipe, even once?”

¶ Sources: National Health and Nutrition Examination Survey and What We Eat in America, U.S. Department of Agriculture. Includes adults aged ≥18 years. The data are estimated from Day 1 dietary recall interviews. The data processing step of adjusting sodium content for salt added during food preparation was discontinued in 2009–2010; equivalent unadjusted estimates for the 2005–2006 and 2007–2008 cycles are based on the default sodium values in the U.S. Department of Agriculture’s Food and Nutrient Databases for Dietary Studies 3.0 and 4.1.

## Footnotes

* NHANES is a complex survey of a multistage probability sample of the civilian, noninstitutionalized U.S. population that combines interviews and physical examinations. During 2005–2012, unweighted response rates ranged from 69.5% to 77.4%. Additional information is available at http://www.cdc.gov/nchs/nhanes.htm.

† Defined as LDL-C fasting values in line with the treatment goals established by the National Cholesterol Education Program (NCEP) Adult Treatment Panel-III (ATP-III) guidelines of <160 mg/dL, <130 mg/dL, and <100 mg/dL for low-, intermediate-, and high-risk groups, respectively. LDL-C was used because it is identified by NCEP as the primary target for lipid-lowering therapy. During 2005–2012, of the 21,858 adults aged ≥18 years interviewed for NHANES, 3,155 were included in the LDL-C analyses.

§ Defined as systolic blood pressure of <140 mm Hg and diastolic blood pressure of <90 mm Hg, based on the average of up to three measurements. Among the participants, approximately 95% had two or three blood pressure measurements during a single physical examination at the mobile examination center. For the 5% with only one blood pressure measurement, that single measurement was used in place of an average. During 2005–2012, of the 21,858 persons aged ≥18 years interviewed for NHANES, 7,591 were included in the blood pressure analyses.

¶ The statistics in this report are estimated from Day 1 dietary recall interviews. The data processing step of “adjusting sodium content for salt added during food preparation” was discontinued in 2009–2010; equivalent unadjusted estimates for the 2005–2006 and 2007–2008 cycles are based on the default sodium values in the United States Department of Agriculture’s Food and Nutrient Databases for Dietary Studies (FNDDS) 3.0 and 4.1. Additional information is available at http://www.ars.usda.gov/ba/bhnrc/fsrg. During 2005–2012, of the 21,858 adults aged ≥18 years interviewed for NHANES, 16,643 were included in the sodium analyses.

** NAMCS is based on a nationally-representative sample of visits to non-federally employed office-based physicians who are primarily engaged in direct patient care. During 2005–2010, unweighted response rates ranged from 58.3% to 61.6%. Additional information available at http://www.cdc.gov/nchs/ahcd/about_ahcd.htm.

†† The percentage of physician office visits to primary care physicians and cardiologists by adult patients aged ≥18 years with ischemic vascular disease (i.e., history of myocardial infarction, angina, stroke, or other vascular diseases) where physicians had prescribed aspirin or other antiplatelet medication. Visits were excluded if the patient had a condition or medication that contraindicated use of antiplatelet medications. Also excluded were obstetrics and gynecology visits. During 2005–2010, of the 149,027 visits by persons aged ≥18 years included in the NAMCS population, 5,657 were included in the aspirin analyses.

§§ The percentage of physician office visits by adult patients aged ≥18 years who screened positive for current tobacco use (i.e., currently smoke cigarettes/cigars or use snuff or chewing tobacco) where tobacco cessation counseling or cessation medications were ordered or provided. During 2005–2010, of the 149,027 visits by persons aged ≥18 years included in the NAMCS population, 17,631 were included in the smoking assessment and treatment analyses.

¶¶ NSDUH is an annual nationwide survey among persons aged ≥12 years. During 2005–2012, weighted response rates ranged from 73.0% to 75.6%. Additional information is available at http://oas.samhsa.gov/nsduh.htm.

*** The percentage of adults aged ≥18 years who reported smoking cigarettes on at least 1 day during the preceding 30 days and ≥100 cigarettes in their lifetime, or who reported smoking cigars or a pipe on at least 1 day during the preceding 30 days. During 2005–2012, of the 304,125 persons aged ≥18 years included in the NSDUH population, 303,523 were included in the current tobacco smoking analyses.

††† Current smokers were defined as those adults aged ≥18 years who had smoked at least 100 cigarettes in their lifetime and now smoke every day or some days. Additional information available at http://www.cdc.gov/nchs/nhis/tobacco/tobacco_statistics.htm.

§§§ Additional information available at http://millionhearts.hhs.gov/docs/mh_smbp.pdf.

¶¶¶ Additional information available at http://www.cdc.gov/tobacco/campaign/tips/about/campaign-overview.html.

**** Additional information available at http://www.thecommunityguide.org.

†††† Additional information available at http://www.cdc.gov/vitalsigns/sodium.
